# Supplementary material for: Effect of Contextualized Versus Non-Contextualized Interventions for Improving Hand Washing, Sanitation, and Health in Rural Tanzania: Study Design of a Cluster Randomized Controlled Trial
Source: Int J Environ Res Public Health. 2019 Jul 15;16(14):2529. doi: 10.3390/ijerph16142529 (PMC6678137; doi:10.3390/ijerph16142529)
Supplement: Supplementary file 1 [file ijerph-16-02529-s001.pdf]

Supplementary materials

Table S1: SPIRIT Guidelines 2013

Section/item	Item No	Description	
**Administrative information**			
Title	1	Descriptive title identifying the study design, population, interventions, and, if applicable, trial acronym	p. 1
Trial registration	2a	Trial identifier and registry name. If not yet registered, name of intended registry	p. 3 + p. 16
	2b	All items from the World Health Organization Trial Registration Data Set	SM9
Protocol version	3	Date and version identifier	Not applicable
Funding	4	Sources and types of financial, material, and other support	p. 2 + p. 17
Roles and responsibilities	5a	Names, affiliations, and roles of protocol contributors	p. 1 + p. 16-17
	5b	Name and contact information for the trial sponsor	p. 17
	5c	Role of study sponsor and funders, if any, in study design; collection, management, analysis, and interpretation of data; writing of the report; and the decision to submit the report for publication, including whether they will have ultimate authority over any of these activities	Not applicable
	5d	Composition, roles, and responsibilities of the coordinating centre, steering committee, endpoint adjudication committee, data management team, and other individuals or groups overseeing the trial, if applicable (see Item 21a for data monitoring committee)	Not applicable
**Introduction**			
Background and rationale	6a	Description of research question and justification for undertaking the trial, including summary of relevant studies (published and unpublished) examining benefits and harms for each intervention	p. 4-5
	6b	Explanation for choice of comparators	p. 4-6
Objectives	7	Specific objectives or hypotheses	p. 5-6

Trial design	8	Description of trial design including type of trial (eg, parallel group, crossover, factorial, single group), allocation ratio, and framework (eg, superiority, equivalence, noninferiority, exploratory)	p. 6

Methods: Participants, interventions, and outcomes

Study setting	9	Description of study settings (eg, community clinic, academic hospital) and list of countries where data will be collected. Reference to where list of study sites can be obtained	p. 6-7
Eligibility criteria	10	Inclusion and exclusion criteria for participants. If applicable, eligibility criteria for study centres and individuals who will perform the interventions (eg, surgeons, psychotherapists)	p. 7
Interventions	11a	Interventions for each group with sufficient detail to allow replication, including how and when they will be administered	p. 8-11
	11b	Criteria for discontinuing or modifying allocated interventions for a given trial participant (eg, drug dose change in response to harms, participant request, or improving/worsening disease)	Not applicable
	11c	Strategies to improve adherence to intervention protocols, and any procedures for monitoring adherence (eg, drug tablet return, laboratory tests)	p. 3 + p. 14-15
	11d	Relevant concomitant care and interventions that are permitted or prohibited during the trial	Not applicable
Outcomes	12	Primary, secondary, and other outcomes, including the specific measurement variable (eg, systolic blood pressure), analysis metric (eg, change from baseline, final value, time to event), method of aggregation (eg, median, proportion), and time point for each outcome. Explanation of the clinical relevance of chosen efficacy and harm outcomes is strongly recommended	p. 12-15
Participant timeline	13	Time schedule of enrolment, interventions (including any run-ins and washouts), assessments, and visits for participants. A schematic diagram is highly recommended (see Figure)	Figure 1
Sample size	14	Estimated number of participants needed to achieve study objectives and how it was determined, including clinical and statistical assumptions supporting any sample size calculations	p. 7-8

Recruitment	15	Strategies for achieving adequate participant enrolment to reach target sample size	Not applicable

Methods: Assignment of interventions (for controlled trials)

Allocation:			
Sequence generation	16a	Method of generating the allocation sequence (eg, computer-generated random numbers), and list of any factors for stratification. To reduce predictability of a random sequence, details of any planned restriction (eg, blocking) should be provided in a separate document that is unavailable to those who enrol participants or assign interventions	p. 8
Allocation concealment mechanism	16b	Mechanism of implementing the allocation sequence (eg, central telephone; sequentially numbered, opaque, sealed envelopes), describing any steps to conceal the sequence until interventions are assigned	p. 8
Implementation	16c	Who will generate the allocation sequence, who will enrol participants, and who will assign participants to interventions	p.7-8
Blinding (masking)	17a	Who will be blinded after assignment to interventions (eg, trial participants, care providers, outcome assessors, data analysts), and how	p. 8, 11, 14-15
	17b	If blinded, circumstances under which unblinding is permissible, and procedure for revealing a participant's allocated intervention during the trial	Not applicable

Methods: Data collection, management, and analysis

Data collection methods	18a	Plans for assessment and collection of outcome, baseline, and other trial data, including any related processes to promote data quality (eg, duplicate measurements, training of assessors) and a description of study instruments (eg, questionnaires, laboratory tests) along with their reliability and validity, if known. Reference to where data collection forms can be found, if not in the protocol	p. 11-15
	18b	Plans to promote participant retention and complete follow-up, including list of any outcome data to be collected for participants who discontinue or deviate from intervention protocols	p.15

Data management	19	Plans for data entry, coding, security, and storage, including any related processes to promote data quality (eg, double data entry; range checks for data values). Reference to where details of data management procedures can be found, if not in the protocol	p. 15
Statistical methods	20a	Statistical methods for analysing primary and secondary outcomes. Reference to where other details of the statistical analysis plan can be found, if not in the protocol	p. 15
	20b	Methods for any additional analyses (eg, subgroup and adjusted analyses)	p. 15
	20c	Definition of analysis population relating to protocol non-adherence (eg, as randomised analysis), and any statistical methods to handle missing data (eg, multiple imputation)	p. 15
**Methods: Monitoring**			
Data monitoring	21a	Composition of data monitoring committee (DMC); summary of its role and reporting structure; statement of whether it is independent from the sponsor and competing interests; and reference to where further details about its charter can be found, if not in the protocol. Alternatively, an explanation of why a DMC is not needed	p. 15
	21b	Description of any interim analyses and stopping guidelines, including who will have access to these interim results and make the final decision to terminate the trial	Not applicable
Harms	22	Plans for collecting, assessing, reporting, and managing solicited and spontaneously reported adverse events and other unintended effects of trial interventions or trial conduct	Not applicable
Auditing	23	Frequency and procedures for auditing trial conduct, if any, and whether the process will be independent from investigators and the sponsor	p. 16
**Ethics and dissemination**			
Research ethics approval	24	Plans for seeking research ethics committee/institutional review board (REC/IRB) approval	p. 16
Protocol amendments	25	Plans for communicating important protocol modifications (eg, changes to eligibility criteria, outcomes, analyses) to relevant parties (eg, investigators, REC/IRBs, trial participants, trial registries, journals, regulators)	Not applicable

Consent or assent	26a	Who will obtain informed consent or assent from potential trial participants or authorised surrogates, and how (see Item 32)	p. 16
	26b	Additional consent provisions for collection and use of participant data and biological specimens in ancillary studies, if applicable	Not applicable
Confidentiality	27	How personal information about potential and enrolled participants will be collected, shared, and maintained in order to protect confidentiality before, during, and after the trial	p. 15
Declaration of interests	28	Financial and other competing interests for principal investigators for the overall trial and each study site	p. 17
Access to data	29	Statement of who will have access to the final trial dataset, and disclosure of contractual agreements that limit such access for investigators	p. 15
Ancillary and post-trial care	30	Provisions, if any, for ancillary and post-trial care, and for compensation to those who suffer harm from trial participation	Not applicable
Dissemination policy	31a	Plans for investigators and sponsor to communicate trial results to participants, healthcare professionals, the public, and other relevant groups (eg, via publication, reporting in results databases, or other data sharing arrangements), including any publication restrictions	p. 16
	31b	Authorship eligibility guidelines and any intended use of professional writers	Not applicable
	31c	Plans, if any, for granting public access to the full protocol, participant-level dataset, and statistical code	Not applicable
**Appendices**			
Informed consent materials	32	Model consent form and other related documentation given to participants and authorised surrogates	SM10
Biological specimens	33	Plans for collection, laboratory evaluation, and storage of biological specimens for genetic or molecular analysis in the current trial and for future use in ancillary studies, if applicable	Not applicable

Table S2: RANAS Questionnaire
**A. RANAS Hand washing**

1. The person who prepares the food, does he/she wash their hands with soap and water before preparing it?
    - Never
    - Seldom
    - Sometimes
    - Most of the time
    - (Almost) always
2. Do you wash your hands with soap and water after toilet use?
    - Never
    - Seldom
    - Sometimes
    - Most of the time
    - (Almost) always
3. What are the direct consequences of diarrhea to you?
    - Loose, watery stool / frequent toilet use
    - Loss of water/ salt from the body
    - Loss of weight/ underweight
    - Fever, weakness, body/ stomach ache
    - Don't know
    - Other
4. Imagine a child has diarrhea, what are the indirect or long-term consequences of diarrhea for that child?
    - High medical costs
    - School Absence
    - Stunting (Low height for age)
    - Malnutrition (Difficulty in taking op food nutrients)
    - Don't know
    - Other
5. What are typical ways you can get diarrhea?
    - Don't wash hands
    - Consume contaminated food (germs, rotten)
    - Consume contaminated drinking water
    - Don't know
    - Other
6. Do you feel there is a risk that you contract diarrhea if you always wash hands with soap and water after contact with stool?
    - Yes
    - No
7. Do you feel there is a risk that you contract diarrhea if you never wash your hands with soap and water after contact with stool?
    - Yes
    - No
8. Do you feel there is a risk that you contract diarrhea if you always wash your hands with soap and water before handling food?
    - Yes
    - No
9. Do you feel there is a risk that you contract diarrhea if you never wash your hands with soap and water before handling food?
    - Yes
    - No

10. Would there be an impact on your daily life if you contract diarrhea?
    - Yes
    - No
11. Would there be an impact on your family's income if you contract diarrhea?
    - Yes
    - No
12. Does it take an effort to always wash hands with soap and water?
    - Yes
    - No
13. Will always washing hands with soap and water prevent you from getting diarrhea?
    - Yes
    - No
14. Is it time consuming to always wash hands with soap and water?
    - Yes
    - No
15. Do you think safe water is expensive?
    - Yes
    - No
16. Do you think soap is expensive?
    - Yes
    - No
17. Do you like washing hands with soap and water?
    - Yes
    - No
18. Does it bother you if you cannot always wash your hands with soap and water after toilet use?
    - Yes
    - No
19. Does it bother you if you cannot always wash your hands with soap and water before preparing/handling food?
    - Yes
    - No
20. Do you like the feeling of clean hands?
    - Yes
    - No
21. How many people in your household always wash hands with soap and water after toilet use?
    - (Almost) nobody
    - Some of them
    - Half of them
    - Most of them
    - (Almost) all of them
22. How many people in your community always wash hands with soap and water after toilet use?
    - (Almost) nobody
    - Some of them
    - Half of them
    - Most of them
    - (Almost) all of them
23. People who are important to you, do they think you should always wash your hands with soap and water?
    - Yes
    - No

24. People in your community, do they think you should always wash your hands with soap and water?
    - Yes
    - No
25. Do you feel a responsibility to yourself to always have clean hands?
    - Yes
    - No
26. In which situations is it critical to wash hands with water and/or soap?
    - After defecating
    - After cleaning a child's bottom
    - After other contact with stool
    - Before breastfeeding a child
    - Before feeding a child
    - Before preparing food
    - Before handling drinking water
    - Before eating
    - Don't know
    - Other
27. Would you wash your hands with soap and water, even when you are in a hurry, for example because your child is crying.
    - Yes
    - No
28. Would you wash your hands with soap and water, even when your (tippy) tap is broken?
    - Yes
    - No
29. Imagine you have stopped washing hands for a few days, for example because there was no water or soap for hand washing. Are you confident that you will start washing hands again?
    - Yes
    - No
30. Do you plan to install a hand washing facility with running water?
    - Yes
    - No
31. Is the hand washing station located at a fixed location?
    - Yes
    - No
32. Will you keep the soap close to the hand washing station?
    - Yes
    - No
33. Do you make sure to always being able to wash your hands?
    - Yes
    - No
34. Imagine that you are in a hurry, for example because your child is crying. Do you have a plan to ensure that you will still be able to wash your hands?
    - Yes
    - No
35. Does it happen that you want to wash your hands with soap and water, but there is no water at home?
    - Never
    - Seldom
    - Sometimes
    - Most of the time
    - (Almost) always

36. How often do you forget to wash your hands after toilet use or before handling food?
    - Never
    - Seldom
    - Sometimes
    - Most of the time
    - (Almost) always
37. Are you committed to always washing your hands with soap and water even if this requires planning, maintenance of a hand washing facility, and a little financial burden for buying soap, water or for repairs?
    - Yes
    - No

B. RANAS Latrine use

1. How often do you use a latrine for urinating and defecating?
    - Never
    - Seldom
    - Sometimes
    - Most of the time
    - (Almost) always
2. Do you feel there is a risk that you contract diarrhea if you always use a latrine?
    - Yes
    - No
3. Do you feel there is a risk that you contract diarrhea if you never use a latrine?
    - Yes
    - No
4. Does it take an effort to always use a latrine?
    - Yes
    - No
5. Will using a latrine prevent you from getting diarrhea?
    - Yes
    - No
6. Is it time consuming to clean a latrine?
    - Yes
    - No
7. Do you think maintaining a latrine is expensive?
    - Yes
    - No
8. Do you like using a latrine?
    - Yes
    - No
9. Does it bother you to defecate in the open?
    - Yes
    - No
10. Does it bother you to use a latrine?
    - Yes
    - No
11. Do you think a latrine smells?
    - Yes
    - No
12. How many people in your household always use a latrine?
    - (Almost) nobody
    - Some of them

- Half of them
- Most of them
- (Almost) all of them

13. How many people in your community always use a latrine?
    - (Almost) nobody
    - Some of them
    - Half of them
    - Most of them
    - (Almost) all of them
14. People who are important to you, do they think you should always use a latrine?
    - Yes
    - No
15. People in your community, do they think you should always use a latrine?
    - Yes
    - No
16. Do you feel a responsibility to yourself to always use the latrine?
    - Yes
    - No
17. Which items are critical for a good latrine?
    - Clean walls
    - Clean surface
    - Sanplat
    - Walls
    - Door
    - Roof
    - Pit
    - Personal cleaning items
    - Respondent didn't know
    - Other
18. Would you use the latrine rather than open defecation, even when you are in a hurry, for example because your child is crying?
    - Yes
    - No
19. Would you repair the latrine if it is damaged after rainy season?
    - Yes
    - No
20. Imagine you have stopped using the latrine for a few days, for example because the latrine was damages. Are you confident that you that you will start using the latrine again?
    - Yes
    - No
21. Do you plan to clean and maintain your latrine at regular times?
    - Yes
    - No
22. Do you make sure to always have a latrine available, for instance when you are in the field or visiting a neighbor?
    - Yes
    - No
23. Do you plan to use a latrine when you are working in the field?
    - Yes
    - No
24. How often does it happen that you want to use the latrine, but that it is too dirty for use?
    - Never

- Seldom
- Sometimes
- Most of the time
- (Almost) always

25. How often do you forget to use the latrine?
    - Never
    - Seldom
    - Sometimes
    - Most of the time
    - (Almost) always
26. Are you committed to always use a latrine, even if this requires planning, cleaning, maintenance and a little financial burden for repairs?
    - Yes
    - No

Table S3: Mini-RANAS intervention

Date	Topic	Norms factor
Nov-Dec 2018	Latrine use:<br>- Personal importance of a good latrine<br>- Being a role model<br>- Public commitment	Personal importance + public commitment
Jan-Feb 2019	Hand washing:<br>- Personal importance of a good hand washing station<br>- Being a role model<br>- When should you wash hands (key times)<br>- Public commitment	Personal importance + public commitment
Mar-Apr 2019	Latrine use + hand washing:<br>- Promote building an improved latrine<br>- Promote building a tippy tap<br>- Distribute contact information for help improving hardware	Others' behavior (+ hardware)
Jun-Jul 2019	Latrine use:<br>- Picture of household head<br>- What is the importance of a clean latrine?	Others' behavior + Others' (dis)approval
Aug-Sep 2019	Hand washing:<br>- Picture of household head<br>- What is the importance of washing hands?	Others' behavior + Others' (dis)approval
Oct-Nov 2019	Latrine use + hand washing:<br>- Movie with local leader encouraging correct behavior	Others' behavior + Others' (dis)approval
Dec-Jan 2019	Hand washing:<br>- Group discussion identifying difficulties and solutions	Others' behavior + Others' (dis)approval
Feb-Mar 2020	Latrine use:<br>- Positive group identity poster<br>- Certificate for their engagement	Positive group identity + public commitment
Apr-May 2020	Repetition	

Table S4: Hand washing Questionnaire

1. Can you please show me where members of your household wash their hands? (Observation)
   - Running water / tippy tap in dwelling
   - Running water / tippy tap in yard or plot
   - Mobile object (bucket/jug/kettle)
   - No hand washing facility available
2. Where is the hand washing station located? (Observation)
   - Near the latrine
   - Near the kitchen/cooking area
   - Elsewhere
3. How long have you had a hand washing station at your home?
   - The hand washing station was installed in the past year
   - 1-5 years
   - 5-10 years
   - Longer than 10 years
4. Which of the following elements are present at the hand washing station? Tick all elements that are present. (Observation)
   - Water and soap
   - Water and ashes
   - Water only
   - Soap or ashes only
   - None of the above
5. Is the hand washing station always operational (water available)?
   - Never
   - Sometimes
   - Most of the time
   - Always
6. How often do you wash your hands per day?
   - Never
   - 1-2 times
   - 3-5 times
   - More than 5 times
7. When do you normally wash your hands? Tick all elements that are relevant.
   - After defecation / using the latrine
   - Before eating
   - Before cooking or handling food
   - Before feeding a child
   - Other
   - Never
8. What do you normally use to wash your hands?
   - Water
   - Water and soap
   - Water and ash
   - Water and soil
   - Nothing
9. Do you pay for water?
   - Yes
   - No
10. Do you have to walk for more than 10 minutes before reaching the water source?
    - Yes
    - No

Table S5: Latrine use Questionnaire

1. Do you have a latrine at home?
    - Yes
    - No
2. When you are at home, where do you defecate?
    - Shared latrine (between 1-4 households)
    - Communal latrine (more than 5 households)
    - None of the above (open defecation)
3. How long have you had a latrine at your home?
    - Less than 1 year
    - 1-5 years
    - 5-10 years
    - Longer than 10 years
4. How many households use the latrine you use?
    - No other households
    - 1-4 Households (shared latrine)
    - More than 5 households (communal latrine)
    - None of the above (open defecation)
5. Exactly how many people use the latrine you use?
6. Does the latrine have a pit? (Observation)
    - Yes
    - No
7. Does the latrine have a slab? (Observation)
    - Yes
    - No
8. Does the latrine have walls? (Observation)
    - Yes
    - No
9. Does the latrine have a roof? (Observation)
    - Yes
    - No
10. Does the latrine have a door or curtain? (Observation)
    - Yes
    - No
11. Does the latrine have a ventilation pipe? (Observation)
    - Yes
    - No
12. Does the latrine have a cover for the pit? (Observation)
    - Yes
    - No
13. Does the latrine have water for flushing? (Observation)
    - Yes
    - No
14. Do you notice any of the following inside the latrine? (observation)
    - Feces on walls or floor
    - Bad smell inside the latrine
    - Many flies
    - None of these
15. Has your pit latrine ever been emptied?
    - Yes
    - No
16. The last time it was emptied, where were the contents emptied?

- Removed using a truck
- Removed using a non-motorized vehicle
- Buried in a covered pit
- Buried in an uncovered pit or open ground
- Other
- Don't know

17. In the past 2 weeks, did you normally use the latrine when defecating at home?
    - Yes
    - No
18. In the past 2 weeks, did you normally use the latrine when defecating elsewhere (work, school, store, bar, …)?
    - Yes
    - No
19. In the past two weeks, when you were at home, did you sometimes defecate in the open?
    - Yes
    - No
20. In the past two weeks, did you sometimes defecate in the open when you were elsewhere (work, school, store, bar, …)?
    - Yes
    - No
21. How often is the latrine cleaned?
    - Never
    - A few times a year
    - Once a month
    - A few times a month
    - Weekly

Table S6: Health Questionnaire

1. How many girls below 5 years of age live in this household?
    a. In the past two weeks, how many of these girls below 5 years had diarrhea?
    b. In the past two weeks, how many of these girls below 5 years had to vomit?
2. How many boys below 5 years of age live in this household?
    a. In the past two weeks, how many of these boys below 5 years had diarrhea?
    b. In the past two weeks, how many of these boys below 5 years had to vomit?
3. How many girls/women above 5 years of age live in this household?
    a. In the past two weeks, how many of these girls/women above 5 years had diarrhea?
    b. In the past two weeks, how many of these girls/women above 5 years had to vomit?
4. How many boys/men above 5 years of age live in this household?
    a. In the past two weeks, how many of these boys/men above 5 years had diarrhea?
    b. In the past two weeks, how many of these boys/men above 5 years had to vomit?

Note: Diarrhea is defined as 2 or more watery stools per day or more frequent than is normal for the household member; Vomiting is defined as the ejection of matter from the stomach through the mouth or nose due to disease. Vomiting due to substance abuse is not included.

5. In the past two weeks, how many children below 5 years of age living in this household have been limited in their activities (school, work, or other daily activities) due to diarrheal illness?
6. In the past two weeks, how many children/adults above 5 years of age living in this household have been limited in their activities (school, work, or other daily activities) due to diarrheal illness?
7. In the past two weeks, how many children below 5 years of age living in this household have sought medical help with a family doctor, general practitioner, traditional healer or other medical doctor due to diarrheal illness?
8. In the past two weeks, how many children/adults above 5 years of age living in this household have sought medical help with a family doctor, general practitioner, traditional healer or other medical doctor due to diarrheal illness?
9. In the past two weeks, how many children below 5 years of age living in this household have been a patient overnight in a hospital or health center due to diarrheal illness?
10. In the past two weeks, how many children/adults above 5 years of age living in this household have been a patient overnight in a hospital or health center due to diarrheal illness?
11. What can you do to protect yourself and others from getting diarrhea? (Multiple answers are possible)
    - Use a latrine
    - Wash hands with water and/or soap
    - Treat drinking water or consume only water from a safe source
    - Eat safe food: Boil, wash, peel, and/or cover food for storage
    - Store drinking water in a container with lid
    - Other
    - Don’t know

Figure S1: EQ-5D-3L

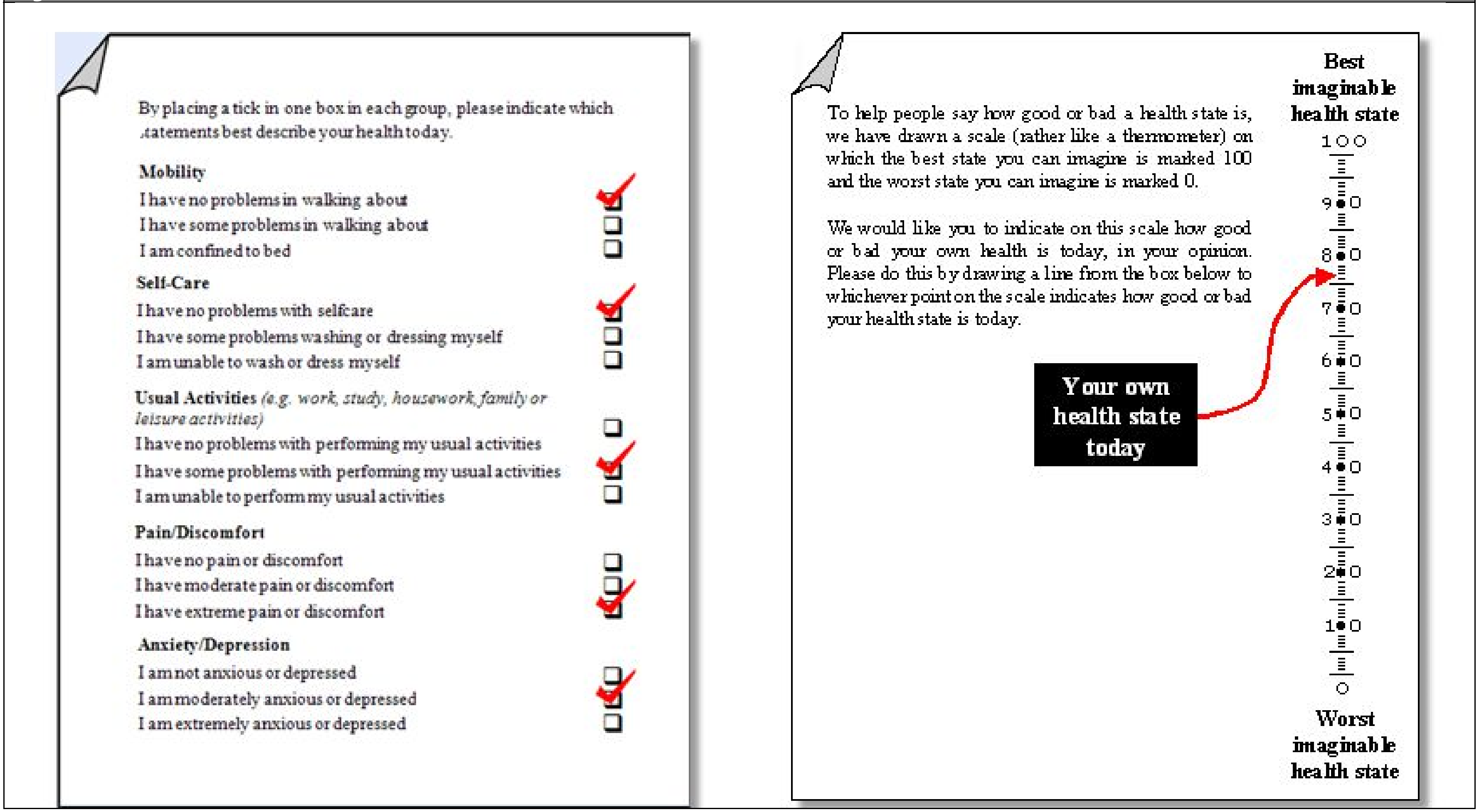

By placing a tick in one box in each group, please indicate which statements best describe your health today.

Mobility
I have no problems in walking about
I have some problems in walking about
I am confined to bed

Self-Care
I have no problems with selfcare
I have some problems washing or dressing myself
I am unable to wash or dress myself

Usual Activities *(e.g. work, study, housework, family or leisure activities)*
I have no problems with performing my usual activities
I have some problems with performing my usual activities
I am unable to perform my usual activities

Pain/Discomfort
I have no pain or discomfort
I have moderate pain or discomfort
I have extreme pain or discomfort

Anxiety/Depression
I am not anxious or depressed
I am moderately anxious or depressed
I am extremely anxious or depressed

To help people say how good or bad a health state is, we have drawn a scale (rather like a thermometer) on which the best state you can imagine is marked 100 and the worst state you can imagine is marked 0.

We would like you to indicate on this scale how good or bad your own health is today, in your opinion. Please do this by drawing a line from the box below to whichever point on the scale indicates how good or bad your health state is today.

Table S7: Demographics questionnaire
Name of data collector<br><br>Household number<br><br>GPS<br>- Latitude<br>- Longitude<br>- altitude<br><br>Village<br>- Buhigwe<br>- Kavomo<br>- Mulera<br>- Songambele<br>- Munyegera<br>- Bulimanyi<br>- Nyamugali<br><br>Subvillages Buhigwe<br>- Lugumba<br>- Buyogwa<br><br>Subvillages Kavomo<br>- Kitulo<br>- Kitagata<br>- Nyandela<br>- Mnyango<br><br>Subvillages Mulera<br>- Lukaro<br>- Rusange<br>- Kamazi<br>- Muzenga<br><br>Subvillages Songambele<br>- Bulambila<br>- Nyamutukula<br>- Bilatenda<br>- Kumsenga<br><br>Subvillages Munyegera<br>- Kabuye<br>- Nyakitanga<br>- Salugale<br><br>Subvillages Bulimanyi<br>- Lulengala<br>- Mudyangoti<br>- Bweru<br>- Buhinda

Subvillages Nyamugali

- Sokoni
- Mbanga
- Nyomvi
- Sakivungwe
- Lukunda
- Kikulazo

Name of household head
Sex of household head
Education of household head

- None
- Adult
- Primary
- Secondary ordinary
- Secondary advanced
- Tertiary vocational
- Tertiary undergraduate
- Tertiary graduate

Education of the wife of the household head

- None
- Adult
- Primary
- Secondary ordinary
- Secondary advanced
- Tertiary vocational
- Tertiary undergraduate
- Tertiary graduate

How many wives does the household head have?

What is your households main source of income?

- Agriculture (own land)
- Livestock
- Running business (wholesale/retail)
- Traditional business (Coal/tailoring)
- Services
- Salaried employment
- Day labor
- Pension
- Other

What is your households monthly average income?

- Less than 30.000 TZS
- Between 30.001-40.000 TZS
- Between 40.001-100.000 TZS
- More than 100.000 TZS
- Don't know

Do you own the land your house is built on?

- Yes

- No

Sex of respondent

- Female
- Male

Age of respondent

What is your relation to the head of the household?

- Self
- Wife
- Husband
- Other

Table S8: WHO registration data set	
**Data category**	**Information**
Primary registry and trial identifying number	Clinicaltrials.gov: NCT03709368
Date of registration in primary registry	October 18, 2018
Secondary identifying numbers	/
Sources of monetary or material support	1. Belgian Red Cross<br>2. Belgian government Directorate-general Development Cooperation and Humanitarian Aid department<br>3. KU Leuven Multi-stakeholders grant
Primary sponsor	Belgian Red Cross
Contact for public queries	Charlotte Schelstraete<br>Phone:<br>E-mail:
Contact for scientific queries	Kim Dockx<br>Phone:<br>E-mail
Public title	Effect of contextualized versus non-contextualized interventions for improving hand washing, sanitation and health in rural Tanzania
Scientific title	Effect of contextualized versus non-contextualized interventions for improving hand washing, sanitation and health in rural Tanzania - cluster randomized controlled trial
Countries of recruitment	Tanzania
Health condition(s) or problem(s) studied<br>Intervention(s)	Hand washing and sanitation behavior, diarrhea<br>- Control cohort: basic intervention + 1 placebo household visit whereby a poster about malaria nets is presented<br>- Contextualized cohort: basic intervention + 9 contextualized WASH household visits<br>- Non-contextualized cohort: basic intervention + 9 non-contextualized WASH household visits<br><br>Definitions used:<br>- Basic intervention: Hardware + CLTS + PHAST<br>- A non-contextualized WASH intervention is a general WASH intervention that is developed beforehand.<br>- A contextualized WASH intervention is a WASH intervention that is adapted according to data collected at baseline to ensure that the intervention optimally fits the needs of the population at hand.
Key inclusion and exclusion criteria	Ages eligible for study: ≥18 years<br>No other eligibility criteria
Study type	Interventional<br>Allocation: randomized<br>Intervention model: parallel assignment

	Masking: single blind (outcome assessor and investigator, but not the implementer or the subject)<br>Primary purpose: prevention
Date of first enrolment	May 2018
Target sample size	1500
Recruitment status	Finished (implementation of intervention is ongoing)
Primary outcome(s)	Hand washing at key times (time frame: baseline vs midline (1year), endline (2 years), and follow-up (3 years))
Key secondary outcomes	Latrine use<br>Health<br>Infrastructure<br>Quality of Life<br>Cost-effectiveness<br>Demographics<br>Compliance

Table S9: Informed consent

Belgian Red Cross Flanders

TANZANIA RED CROSS SOCIETY

Design	Cluster randomized controlled trial
Title	Effectiveness of different software interventions for improving hand washing, sanitation, water storage and water transport in rural Tanzania: an impact evaluation
Funding	Belgian Red Cross-Flanders (BRC-Fl)
Collaboration	Tanzania Red Cross Society (TRCS)
Approval	This study was approved by the National Institute for Medical Research (NIMR) of Tanzania.

Dear Sir or Madam,

You are invited to participate in a research study conducted by Belgian Red Cross-Flanders and Tanzania Red Cross Society. The purpose of this research is to determine the effectiveness of different software interventions for improving hand washing, sanitation, water storage and water transport in rural Tanzania.

What will you be asked to do?

If you consent to participate, you will be ***randomly assigned to one of three interventions***. Each intervention arm will receive opportunities to improve hardware (latrines, hand washing stations, etc.), and to inform themselves about better water, sanitation and hygiene behavior. One study arm will also receive first aid information.

The intervention is spread over a ***3 year period (June 2018 – May 2021).*** We encourage you to participate in as many community meetings and household visits as possible. You will be assessed 5 times to investigate whether the intervention has improved your situation, namely before the start of the intervention, after 1 year, after 2 years, after 3 years, and 6 months after the intervention has finished. This will allow us to monitor your progress. Each assessment will take approximately 2 hours. The assessment will consist of an interview and spot checks.

Risks

The interventions and assessments are harmless. There are no known risks associated with participation in this study.

Benefits

We expect the project to benefit you by improving WASH hardware and increasing WASH knowledge and/or first aid knowledge. We hope that these improvements will reflect in a better health.

It is hoped that the results of this study will improve our understanding of different software interventions and how these may affect behavior. The outcomes of the study may have important consequences for future WASH programs. As incorrect WASH behavior has an enormous health and economic burden, evaluating the most effective WASH intervention could reduce health care costs substantially.

Compensation

There is no financial compensation for participating in this study.

Confidentiality and privacy

Any information collected is confidential. Your name will not be kept on any documents except a participant key. A pseudonym will be used to protect your identity. Your name or other identifying information will not be reported in any publications. The key linking your name to the data will be destroyed after data analysis is complete.

Belgian Red Cross Flanders

TANZANIA RED CROSS SOCIETY

**Voluntary**
Participation is voluntary. The study will not incur costs for the participant. If you are uncomfortable for any reason and wish to discontinue the experiment, please inform the facilitators and/or assessors. You are free to stop at any time for whatever reason, and will not be penalized for choosing to end the experiment.

I do hereby consent to take part in the research study. I have been properly informed about the design, methods and objective of the study. Any questions I had have been answered satisfactorily.

I am aware that I may stop at any time for whatever reason, and will not be penalized for choosing to end the experiment. My signature below shows that I understand and agree with all of these statements.

Name participant: ....

Date: ....

Signature:

Name provider: ....

Date: ....

Signature:

**Principal investigator contact:**	Local contact:
Kimberly Dockx<br>Researcher, CEBaP, Belgian Red Cross-Flanders<br>Motstraat 42 2800 Mechelen, Belgium<br>TEL. +32 15 44 35 81<br>Kimberly.dockx@cebap.org	An Vanderheyden<br>Tanzania Red Cross Society<br>Mwai Kibaki Road Plot 53 Block C Dar Es Salaam<br>TEL. +255 682 13 27 28<br>An.vanderheyden@rodekruis.be

For any complaints or other concerns regarding the ethical aspects of this study, please contact

National Institute for Medical Research (NIMR), Tanzania:
info@nimr.or.tz | TEL. +255 222 12 14 00

Social and Societal Ethics Committee (SMEC), KU Leuven, Belgium:
smec@kuleuven.be
